# CoNVEX: copy number variation estimation in exome sequencing data using HMM

**DOI:** 10.1186/1471-2105-14-S2-S2

**Published:** 2013-01-21

**Authors:** Kaushalya C Amarasinghe, Jason Li, Saman K Halgamuge

**Affiliations:** 1 Department of Mechanical Engineering, University of Melbourne, Parkville, VIC 3010, Australia; 2 Bioinformatics Core Facility, Peter MacCallum Cancer Centre, VIC 3002, Australia

**Keywords:** CNV detection, Cancer Genome, Targeted resequencing, Whole exome sequencing, Hidden Markov Models, Discrete Wavelet Transform

## Abstract

**Background:**

One of the main types of genetic variations in cancer is Copy Number Variations (CNV). Whole exome sequenicng (WES) is a popular alternative to whole genome sequencing (WGS) to study disease specific genomic variations. However, finding CNV in Cancer samples using WES data has not been fully explored.

**Results:**

We present a new method, called CoNVEX, to estimate copy number variation in whole exome sequencing data. It uses ratio of tumour and matched normal average read depths at each exonic region, to predict the copy gain or loss. The useful signal produced by WES data will be hindered by the intrinsic noise present in the data itself. This limits its capacity to be used as a highly reliable CNV detection source. Here, we propose a method that consists of discrete wavelet transform (DWT) to reduce noise. The identification of copy number gains/losses of each targeted region is performed by a Hidden Markov Model (HMM).

**Conclusion:**

HMM is frequently used to identify CNV in data produced by various technologies including Array Comparative Genomic Hybridization (aCGH) and WGS. Here, we propose an HMM to detect CNV in cancer exome data. We used modified data from 1000 Genomes project to evaluate the performance of the proposed method. Using these data we have shown that CoNVEX outperforms the existing methods significantly in terms of precision. Overall, CoNVEX achieved a sensitivity of more than 92% and a precision of more than 50%.

## Background

Commercial products of Next Generation Sequencing (NGS) Technologies such as Roche/454 FLX, Illumina Genome Analyzer/HiSeq, Applied Biosystems SOLiD™System and Helicos Heliscope™have enabled the sequencing of DNA much faster and cheaper than before [1]. These have shifted the paradigm of biological sequence analysis to a new level. Currently these are not only being used for the sequencing of whole genome, but also for sequencing of known exons and transcriptomes as well. The main motivations behind the technology of targeted resequencing (TR) include the following among others. The actual coding regions or the exons of the human genome account only for ~1% of the total sequences, which consequently gives about 30 Mb data compared to 3 Gb data in WGS [2]. Currently, getting higher coverage of targeted regions using NGS technologies is about six times [3] cheaper and faster compared to achieving the same coverage of whole genome. On the other hand approximately 85% of disease causing mutations lie in the coding regions [4]. Targeted resequencing has been mainly used in medical sequencing to find disease causing genetic variations (a review can be found in [5]). Recent studies on TR and WES data have successfully detected cancer specific mutations (somatic mutations) in breast cancer [6-8], ovarian cancer [8] and prostate cancer [9]. Although, exome sequencing has been successfully used to find small variations in cancer genomes, its potential to find large structural variations such as CNV has not yet been fully explored.

Cancer arises due to the acquisition of many somatic variations by the DNA of cancer cells [10]. Copy Number Alterations (CNA) play a major part in the progression of this deadly disease [11]. Until recently, the most common method to detect CNV in cancer DNA was to use micro array based technologies. However, during last 2 - 3 years many algorithms have been developed to identify CNV in cancerous data generated by whole genome sequencing [11-15], making use of the vast amount of data produced by NGS technology. The higher resolution that can be achieved through NGS data has helped to detect new variations that were undetectable previously and include CNVs which are as small as 50 bp [16]. These methods use the number of reads mapped to a particular region in the genome, to find copy number varying regions in one genome compared to one or more other genomes. Some of these methods have been adapted from the methods used in aCGH. For example Circular Binary Segmentation (CBS) [17] and Hidden Markov Model (HMM) [18]. However, methods in whole genome sequencing cannot be directly applied to whole exome sequencing data due to the small size and sparseness of these data [19]. On the other hand, the useful signal will be hindered by the intrinsic noise present in the exome sequencing data itself due to various biases introduced in target capturing and sequencing phases. To address these issues and to utilize the advantages provided by targeted resequencing, new algorithms have to be developed. Since the end of 2011, very few bioinformatics methods for detecting copy number variations in targeted resequencing data have been published. The method in [20] describes the use of TR data to detect CNV in cancer samples. However, the targeted regions in this method are larger in size (~ 40 kb), where as exons are much shorter (200 bp -300 bp). Methods such as [21,22] are developed to find CNV in non cancerous exome data, such as in population studies. CONTRA [19] is a recent method proposed to evaluate cancer TR data using a pooled or a matched normal sample. ExomeCNV [23] and Var Scan 2 [24] are specifically designed for whole exome sequencing of cancer samples. A limitation in these approaches is that they have a higher number of false positives which result in a very low precision (further discussed in Results and discussion section for ExomeCNV).

In this work, we present CoNVEX, a method that evaluates exon level depth of coverage ratios to assess variation in copy number of whole exome capture data produced from cancer samples. We propose to use Discrete Wavelet Transformation denoising to reduce the variability of coverage ratios and then use HMM to detect copy number variations. Our method reduces the number of false positives by efficient pre-processing of the data, which results in a mean precision of more than 50%.

## Methods

### Data pre-processing

#### Depth of coverage ratios at each targeted region

Number of reads covering each base at a targeted region is calculated using BEDTools [25]. Then the exon level depth of coverage (DOC) is calculated as mean of the per base coverage of that particular exon. To control the quality, only the regions having more than 10 bp DOC in the control sample are retained for further analysis. To correct for the differences in total number of reads in tumour and control samples, the exon level DOC is divided by the mean of DOC of all the exons in that sample. Then the exon level DOC ratio at region *i *is calculated as,

(1) $$R_{i}=\frac{N_{T_{i}}}{N_{C_{i}}}$$

Where $N_{T_{i}}$ and $N_{C_{i}}$are the mean normalized DOC of tumour and control respectively.

#### DWT smoothing of the data

The actual copy number of the exon regions can be masked by the noise present in the data itself. This would lead to lot of false positives. The raw signal of exon level ratios can be represented as below,

$$R_{i}={\bar{R}}_{i}+\epsilon_{i}$$

Here, ${\bar{R}}_{i}$ is the true signal of copy number variation with additive noise, *ϵ_i_*. This noise can be assumed to be iid with *N*(0, *σ*^2^)where *σ *is the standard deviation of the distribution. We have used DWT smoothing [26] on *R_i_*, to detect true signal ${\bar{R}}_{i}$ to increases the ability of actual copy number prediction. The DWT smoothing procedure starts by first taking discrete wavelet transformation of ratios using "HAAR" wavelet. The fundamental assumption behind discrete wavelet transform is that, there is a correlation between the two neighbouring samples or data points. This is very much true in predicting CNVs as they span multiple successive exons. The selection of HAAR wavelet family was based on the fact that it computes the wavelet coefficients as the difference between two near by blocks of data points. This feature helps to retain the information regarding copy number aberration points. The shrinking of the DWT coefficients were done using soft thresholding function and the threshold value was calculated by Stein's unbiased risk estimator (SURE) for each level of DWT. Finally, the modified coefficients were used to reconstruct the de-noised signal at *i^th^*location of chromosome *j*, ${\bar{R}}_{ij}$, by taking the inverse transform.

### CNV prediction using a Hidden Markov Model

The copy number state for each targeted region is assigned using a Hidden Markov Model. The copy numbers are represented by the hidden states and as default we have used states from 0 to 5. These six states can be interpreted in biological context as homozygous deletion (copy 0), hemizygous deletion (copy 1), no CNV or copy neutral (copy 2), 1 copy gain (copy 3), 2, and 3 copy amplification (copy 4 and 5). DWT smoothed ratios, ${\bar{R}}_{ij}$, are fed to the model as observations. Each chromosome *j *of each tumour-control samples pair is considered separately for copy number identification using the HMM. The fitted discrete time HMM is given below with the same notations as described by Rabiner [27] and Fridlyand et.al. [18].

1. The total number of hidden states in the model is given by *K *and those are denoted by *S *= *S*_1_, *S*_2_,..., *S_K_*. If there are *L *exons in the sample of consideration, the state of *l^th^*exon (*e_l_*) equals to *S_k_*where 1 ≤ *l *≤ *L *and 1 ≤ *k *≤ *K*.

2. The initial state distribution *π *= {*π_k_*} where

$$\pi_{k}=P\left(e_{1}=S_{k}\right),\;1\leq k\leq K$$

3. The state transition probability distribution *A *= *a_mp_*where

$$a_{mp}=P\left(e_{l+1}=S_{p}|e_{l}=S_{m}\right),\;1\leq m,p\leq K$$

4. The emission probability distribution is given by *B *= {*b_k_*(**O**)} where

$$\left\{b_{k}\left(O\right)\right\}=N\left(O_{l},\mu_{k},\sigma^{2}\right),\;1\leq l\leq L\;\;and\;\;1\leq k\leq K$$

Here, N  represents the Gaussian distribution. Mean (*μ_k_*) of that distribution vary with different states and the provided normal cell contamination percentage and ploidy. We used a common standard deviation, *σ*, to all states.

The above HMM can be represented compactly as *λ *= (*A, B, π*) where *A, B *and *π *represent transition probability matrix, emission probability distribution and initial state distribution. When fitting the above HMM, the *K *states must be fixed at first and normal contamination and tumour ploidy must be given as inputs.

The optimal *λ *is selected by optimizing the negative log-likelihood [27,28]. The initial state distribution *π *is chosen such that higher probability is attached to the most abundantly expected state or the normal state (i.e. copy 2 in normal humans). Similarly, the transition probability matrix *A*, is chosen such that, a higher probability is assigned to remain in the same state and lower probability is assigned to transition to another state. Also the transition to normal state has higher probability than transition to a CNV state. Then we used Viterbi Algorithm to assign the most appropriate copy number state for each exon.

#### Relationship between DOC ratio and copy number

Without any imperfections, the normalized ratios between regional DOC of tumour and control samples $\left({\bar{R}}_{ij}\right)$ should reflect the relative copy numbers of the regions in tumour sample compared to control sample. For example, the ratios (0, 0.5, 1, 1.5, 2) correspond to the relative copy numbers (0, 1, 2, 3, 4). With no normal cell admixture and existence of a diploid cancer genome, these ratios would be the mean of emission distributions that belonged to hidden states of HMM described above. In the presence of normal cell admixture and anueploidy, the ratios would become,

(2) $${\bar{R}}_{ij}=\frac{\alpha P_{C_{ij}}+\left(1-\alpha \right)P_{T_{ij}}}{P_{C_{ij}}}$$

where *α *is the normal cell contamination in tumour cells, $P_{C_{ij}}$ is the ploidy of normal cells which equals to 2 in diploid human genome, and $P_{T_{ij}}$ is the ploidy of tumour cells. As proposed by Fidlyand et.al. [18], by performing median normalization on (2), the ratios will depend on tumour ploidy only. After performing median normalization, the ratio is given by

(3) $$\begin{array}{ccc} \rho_{ij}= & \frac{\alpha P_{C_{ij}}+\left(1-\alpha \right)P_{T_{ij}}}{P_{C_{ij}}}/median\left(\frac{\alpha P_{C_{ij}}+1\left(1-\alpha \right)P_{T_{ij}}}{{P_{C}}_{ij}}\right) & \\ = & \frac{\alpha P_{C_{ij}}+\left(1-\alpha \right)P_{T_{ij}}}{median\;{\left(\alpha P_{C_{ij}}+\left(1-\alpha \right)P_{T_{ij}}\right)}^{}}\\ = & \frac{\alpha P_{C_{ij}}+\left(1-\alpha \right)P_{T_{ij}}}{P_{T}} \end{array}$$

where *P_T _*is the most abundant ploidy in the tumour sample.

### Data from 1000 Genome Project

We randomly selected six samples, NA18536, NA18543, NA18544, NA18548, NA18557, NA18558, from 1000 Genome project, which share some common attributes, to evaluate the performance of the proposed method. These selected individuals have been studied by the HapMap project http://www.hapmap.org. The common features in these individuals are (i) exome sequencing was performed by the Beijing Genome Institute, hence a common exome-capture (NimbleGen V2) has been performed, (ii) male individuals and (iii) from CHB population.

### Simulated data with known copy number variations

We used depth of coverage data at each exon of 1000 Genome samples to simulate CNV. This ensures that we retain as much intrinsic noise present in non copy number varying regions. The simulation procedure is as follows,

1. First, we retain only the copy number neutral regions in each sample. The CNV information were downloaded from the HapMap project genotype file.

2. We selected one sample (NA18536) as the Control and others as the tumour with known CNV.

3. To do the simulation of gains and losses we randomly selected a region in the Chr 1 and reduce (e.g: multiplied by 0.05) or amplified (e.g: multiplied by 2) the number of reads in that particular region. For each variation type, we perform 100 simulations.

4. When we evaluated the performance using only one sample (NA18543), we used 100 simulations for each variation type. When we used 5 samples in simulations, 20 variations were simulated in each individual sample.

5. To incorporate contamination in the simulation, we mix the control sample and simulated sample as per the relationship (*α*∗ *Control *+ (1 - *α*) ∗ *Tumor*) where *α *is the contamination proportion.

## Results and discussion

### Exon level depth of coverage ratios to detect CNV in whole exome data

**Figure 1:**
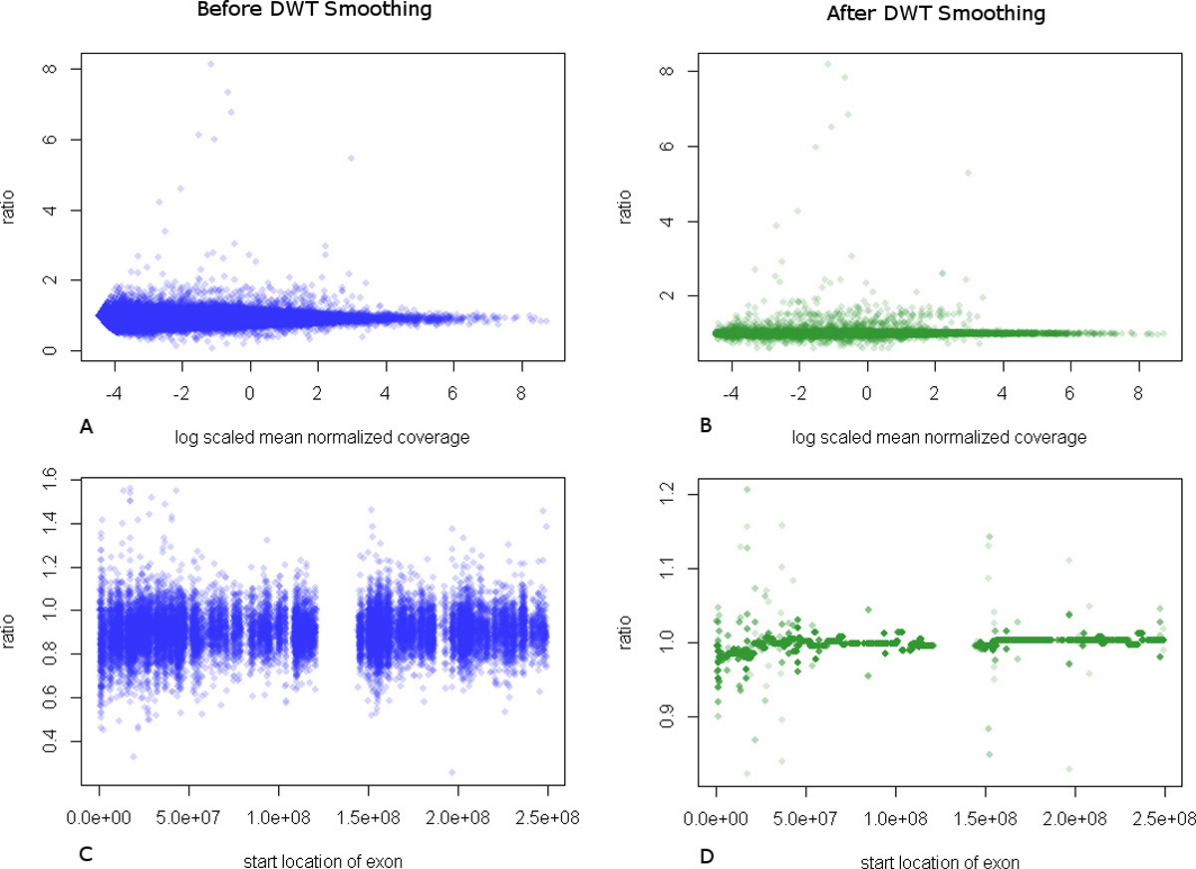
**Exon level coverage ratios before and after smoothing**. Exon level coverage ratios among tumour and matched normal samples (A, C) before DWT smoothing and (B, D) after DWT smoothing. (A, B) show the ratios against the mean log coverage among the two samples. (C, D) show ratios of chromosome 1 exons against their start locations.

We have used normalized depth of coverage ratios of the exons among tumour/normal pair to identify the underlying copy number losses and gains. As a quality control procedure, all the regions in matched normal sample, with less than an average coverage of 10 are eliminated in both tumour and normal data sets. However, the useful signal to be used in CNV detection is depleted by the noise present in data itself. This can be attributed to the GC content bias, mappability, bait capture bias [20] etc. In line with the observations made in [19], we observed that variation in exon level DOC ratios depends on the average coverage of both tumour and normal samples (Figure 1A). This introduces higher variation in ratios in lower coverage levels.

Different methods have been proposed to reduce the experimental biases present in TR data. These include GC content bias reduction using regression methods [20,21], taking base level ratios between normal and control samples [19,23] and bait capture bias reduction using log transformation [20]. Those methods, adapted from aCGH or whole genome sequencing based approaches, try to reduce different experimental biases separately. Hsu et. al. [29] proposed DWT smoothing as an effective method to extract true copy number variations from aCGH data.

In this work, we propose to combine the strengths of both DWT and HMM to robustly predict copy number variations in cancer samples. The main novelty of our approach is the use of DWT smoothing to reduce experimental biases present in whole exome sequencing data prior to applying a Hidden Markov Model. These experimental biases are modelled here as additive noise to the true signal. The wavelet coefficients, which are the differences between two nearby data blocks, can be used to reduce noise. This is achieved through approximating some coefficients that do not by pass a certain threshold to zero. After thresholding step when the inverse transform is performed on these wavelet coefficients, we can generate a smoother version of the input signal. Exon level ratios, before and after DWT smoothing, for data downloaded from 1000 Genome project (http://www.1000genomes.org) are given in Figure 1.

After smoothing, we applied an HMM described in Methods section to detect copy gains and losses. Hidden Markov Models have been previously used to detect CNV in exome data (an R package called ExomeCopy) [21], but not used in this manner to detect CNV in tumour samples. The differences between ExomeCopy and CoNVEX are,

• ExomeCopy uses HMM to identify CNVs in male patients with X-linked Intellectual Disabilities (XLID)

• They have used depth of coverage of exons as observations or emissions of hidden states

• The robustness in copy identification is achieved by pooling coverage data from all patients

Therefore, it fails to identify relative copy number in cancer samples against a matched normal.

### Comparison of the performance of CoNVEX against other methods

#### Comparison against ExomeCNV using simulated data

We carried out a comparison between the proposed method and the existing method, ExomeCNV [23]. Using simulated data, we were able to assess the performance of CoNVEX and ExomeCNV for different size ranges.

A true positive (TP) is identified when the gain or loss of an exon is correctly identified by the algorithm and a false positive (FP) identification is defined in the same manner. When using ExomeCNV, we used their primary CNV detection method (here after referred to as ExomeCNV1) and the extension which combines DNACopy [17] (here after referred to as ExomeCNV2) separately on our simulated data sets. The DNACopy version of ExomeCNV is applied to make sure that we get results for all exons that pass the default cut-off level of the coverage (this is a direction given by the authors of the paper). We used default parameters given in ExomeCNV R package for CNV prediction, except for read length and admixture rate, which we set to 90 and 0.0 in our evaluation.

We used simulated data as described in Methods section to carry out the comparison. For this, we simulated deletions and duplications in different size ranges. The results of this evaluation are given in Table 1, 2, 3. Both CoNVEX and ExomeCNV2 perform better compared to ExomeCNV1 in detecting deletions and duplications. This shows that detecting variations by segmenting the exome works well, rather than only considering one exon at a time and depicting its copy number when there are large variations. Another note regarding ExomeCNV1 is that it doesn't produce results for about 16% of the exons in the whole exome.

**Table 1:** Performance of proposed method for 100 simulations.

Type	Proposed Method			
	
	Sensitivity	Specificity	Precision	Accuracy
Deletions (1 k -1 M bp)	97.82 ± 12.37%	99.94 ± 0.081%	79.25 ± 23.23%	99.94 ± 0.081%

Duplications (1 k -1 M bp)	95.25 ± 19.64%	99.93 ± 0.082%	77.04 ± 26.43%	99.93 ± 0.085%

Performance of CoNVEX in terms of sensitivity, specificity, recall and accuracy. We listed mean and the standard deviation of the each performance measure.

**Table 2:** Performance of ExomeCNV1 for 100 simulations.

Type	ExomeCNV1			
	
	Sensitivity	Specificity	Precision	Accuracy
Deletions (1 k - 1 M bp)	97.91 ± 2.81%	86.20 ± 1.57%	8.76 ± 6.54%	86.24 ± 1.56%

Duplications (1 k - 1 M bp)	90.68 ± 9.02%	86.26 ± 1.55%	8.96 ± 8.57%	86.28 ± 1.54%

Performance of ExomeCNV in terms of sensitivity, specificity, recall and accuracy. These results are obtained from running the primary method of ExomeCNV. Each point indicates mean and standard deviation of the measure.

**Table 3:** Performance of ExomeCNV2 for 100 simulations.

Type	ExomeCNV2			
	
	Sensitivity	Specificity	Precision	Accuracy
Deletions (1 k - 1 M bp)	99.26 ± 2.11%	96.00 ± 1.67%	8.69 ± 6.50%	96.01 ± 1.66%

Duplications (1 k - 1 M bp)	99.98 ± 0.16%	96.06 ± 1.65%	9.62 ± 9.25%	96.08 ± 1.64%

Performance of ExomeCNV in terms of sensitivity, specificity, recall and accuracy. These results are obtained from running the extension of ExomeCNV which includes DNACopy package. Each point indicates mean and standard deviation of the measure.

When compared with ExomeCNV2, our method showed superior performance in terms of specificity, precision and accuracy. Slight decrease in sensitivity was observed in CoNVEX, this is mainly due to the detecting short variations involving 1 or 2 exons. This can be attributed to the smoothing step we performed using DWT. Because of this we separately tested the performance of CoNVEX for shorter variations sizes as described below. Both versions of ExomeCNV, showed very poor performance when it comes to precision, as it tries to detect as many as possible variations to maintain a higher sensitivity rate.

#### Performance assessment of other methods against CoNVEX

To evaluate the performance of CoNVEX against VarScan2 [24], ExomeCopy [21] (uses an HMM to identify CNV) and CONTRA [19], we used actual genotype of chromosome 1 in NA18543 individual (test) against NA18536 individual (control). All methods were run using their default settings. The results are given in Table 4.

**Table 4:** Performance of CoNVEX against other methods.

Method	True positives	False positives
CoNVEX	9/10	10/15850

Var Scan2	6/7	4983/15283

ExomeCopy	0/10	9/15850

CONTRA	0/10	0/15847

Table shows the number of exons (numerator) that have been identified as true positives and false positives by each method. The denominator shows the total true positives (2*^nd^*column) or true negatives (3*^rd^*column). Total number of true negatives differ among methods due to filtering and quality control done by each of them.

ExomeCopy and CONTRA did not identify any of the variations present in the test sample. This can be attributed to the fact that these are specifically designed for using a background sample [21] or a robust baseline [19]. VarScan 2 was able to identify the hemizygous duplication in the region with 60% sensitivity, however the number of false positives reported by the method was very high (false positive rate of 32%). CoNVEX performed well with 90% sensitivity and 0.06% false positive rate.

### Performance of proposed method at different duplication and deletion sizes

**Figure 2:**
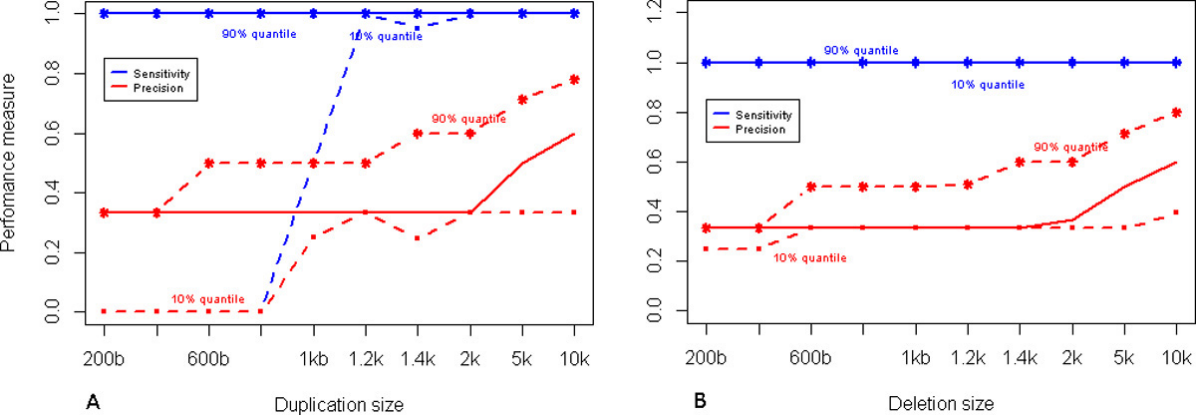
**Performance at different variations sizes**. Performance of CoNVEX when detecting short variations. Performance is measured by sensitivity and precision. Both graphs show median (solid lines), 0.1 quantile (dashed lines) and 0.9 quantile (dashed lines) of the results from 100 simulations of (A) duplication and (B) deletions. The solid blue line shows the median sensitivity and the solid red line shows the median precision. The sizes considered are 200, 400, 600, 800, 1 k, 1.2 k, 1.4 k, 2 k, 5 k and 10 k bases.

We observed that small deletions or duplications only span one exon and at most 2 exons due to the sparseness of the exome data. To evaluate the performance of CoNVEX in short variation sizes, we carried out a performance assessment using simulated data of small deletions and duplications in chromosome 1 of NA18536 and NA18543 individuals. The results are given in Figure 2.

Median sensitivity of CoNVEX for small variation detection is 100%. Every deletion of size, more than 200 bp was detected by our method. Hence, giving a mean sensitivity of 100% for detecting deletions. Mean sensitivity of detecting each duplication size was more than 85%. As seen in the graph, almost every variation of size of more than 800 bases can be detected by the proposed method. Also, a median precision of more than 30% can be achieved.

### Performance assessment at different levels of contamination

**Figure 3:**
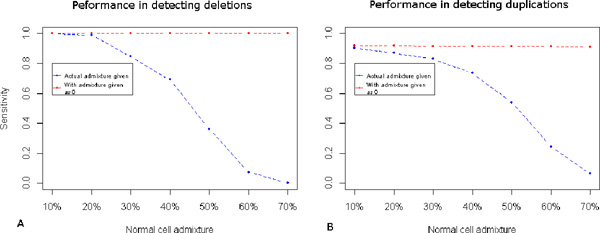
**Performance of CoNVEX at different admixture rates**. The plots show mean sensitivity of CoNVEX at different admixture rates for (A) duplications and (B) deletions. The dashed red line shows the sensitivity when, user provides the admixture rate as an input. The dashed blue line shows the sensitivity of the model when it expects zero admixture. The size range of duplications and deletions considered here is 1 k -10 k bp.

Normal cell admixture in cancer sample is an issue that has to be taken into account when predicting copy number losses and gains. The presence of admixture shrinks the DOC ratios to 1 (also discussed in Methods). Our method works on the assumption that the user will provide the contamination percentage as an input. However, these data might not be available for every experiment. Hence, we carried out an evaluation of our method based on simulated data from NA18543 for two scenarios. First scenario was to consider the availability of admixture rate and second was to run the programme without any indication of contamination. The performance of CoNVEX, for admixture rates ranging 10% to 70%, in terms of sensitivity, under the first scenario is given in Figure 3A and the second scenario in Figure 3B. This admixture rate range is normally present in cancer samples [23]. The performance of the method drastically reduces after 50% contamination in scenario 1. However, if proper estimation of admixture rate is provided, we didn't see much difference in the performance level of CoNVEX.

## Conclusions

Exome sequencing data can be used to detect copy number variations as an initial screening procedure. It is a cheap and time efficient method. We have successfully applied the proposed method on exome data to identify CNVs spanning one to thousands of exons. However, actual breakpoint of the CNV would not necessarily lie in the coding region. This limits the use of WES in identifying actual breakpoints of the CNV.

As discussed in the Results and Discussion section, we have achieved a higher precision than existing methods in detecting variations due to the data smoothing step. However, detection of some of the small variations may be missed by this smoothing step, as these can be recognised as noise. Further analysis is needed in order to better detect these variations among higher level of noise.

Although, we have used a matched normal sample to detect CNVs, the CNV identification can be done based on a pooled normal sample as described in [19]. This might give an advantage in finding CNVs in familial studies assuming all members have a median copy number of two.

## Competing interests

The authors declare that they have no competing interests.

## Authors' contributions

KCA designed the method, evaluated the performance and drafted the manuscript. JL and SKH contributed to improve the method. All authors read and approved the final manuscript.
