# Correction: CoNVEX: copy number variation estimation in exome sequencing data using HMM

**DOI:** 10.1186/1471-2105-14-S2-S26

**Published:** 2013-06-03

**Authors:** Kaushalya C Amarasinghe, Jason Li, Saman K Halgamuge

**Affiliations:** 1 Department of Mechanical Engineering, University of Melbourne, Parkville, VIC 3010, Australia; 2 Bioinformatics Core Facility, Peter MacCallum Cancer Centre, VIC 3002, Australia

## 

Our proposed method, to detect copy number variations in whole exome sequencing data [1] was published in BMC Bioinformatics as a special issue containing the proceedings of The Eleventh Asia Pacific Bioinformatics Conference (APBC 2013). After the publication, it was brought to our attention that name of our software has a conflict with another software developed by a project carried out at Wellcome Trust Sanger Institute (http://www.uk10k.org/assets/ashg_vijayarangakannan_etal_2012.pdf). We were not aware of this project when we first named our method as "CoNVEX", in mid 2012. Therefore, we would like to thank Dr. P. Vijayarangakannan, one of the developers of the other method, for bringing this to our attention. We would like to mention that, although both software share the same name, they are different in terms of computational methods and types of exome sequencing data used.

To avoid any confusion associated with the software name, we would no longer call our software as "CoNVEX". Our software will be further developed with a new project name, "Aberration Detection in Tumour Exome (ADTEx)".
